# Supplementary material for: Tailoring sodium intercalation in graphite for high energy and power sodium ion batteries
Source: Nat Commun. 2019 Jun 13;10:2598. doi: 10.1038/s41467-019-10551-z (PMC6565630; doi:10.1038/s41467-019-10551-z)
Supplement: Supplementary file 1 — Supplementary Information [file 41467_2019_10551_MOESM1_ESM.pdf]

Supplementary Information for

Tailoring sodium intercalation in graphite for high energy and power sodium ion batteries

Xu *et al.*

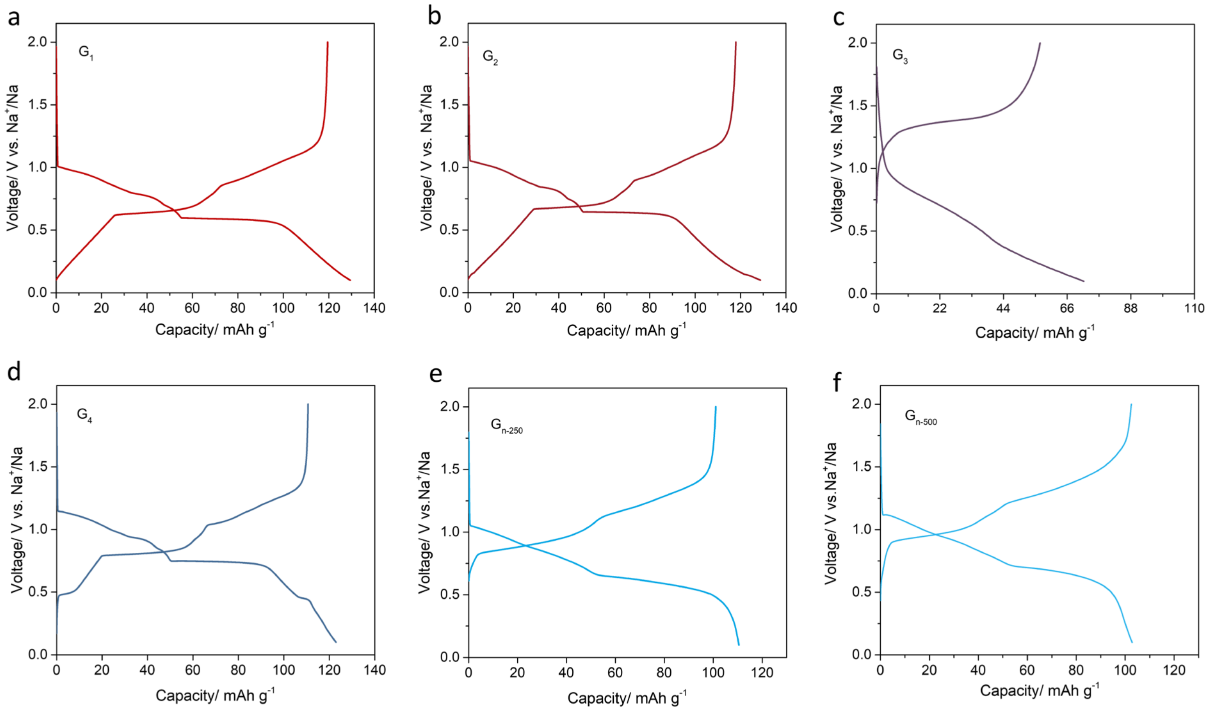

Supplementary Figure 1 | Voltage profiles of graphite cycled in different electrolytes. 1 M $NaPF_6$ (a) G_1, (b) G_2, (c) G_3, (d) G_4, (e) G_{n-250} and (f) G_{n-500} electrolyte, respectively, at 50 mA g^{-1}.

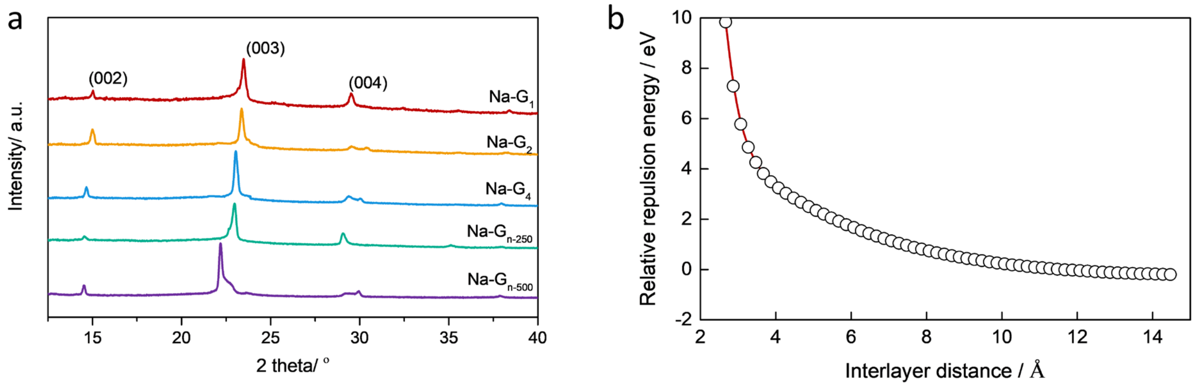

Supplementary Figure 2 | Structures of fully co-intercalated graphite. (a) XRD patterns of fully sodiated graphite in G_1, G_2, G_4, G_{n-250} and G_{n-250} systems, (b) relative repulsion energy between charged graphene layers. The amount of charge transfer is set to one per C_{20}. Repulsion energies are normalized to C_{20}.

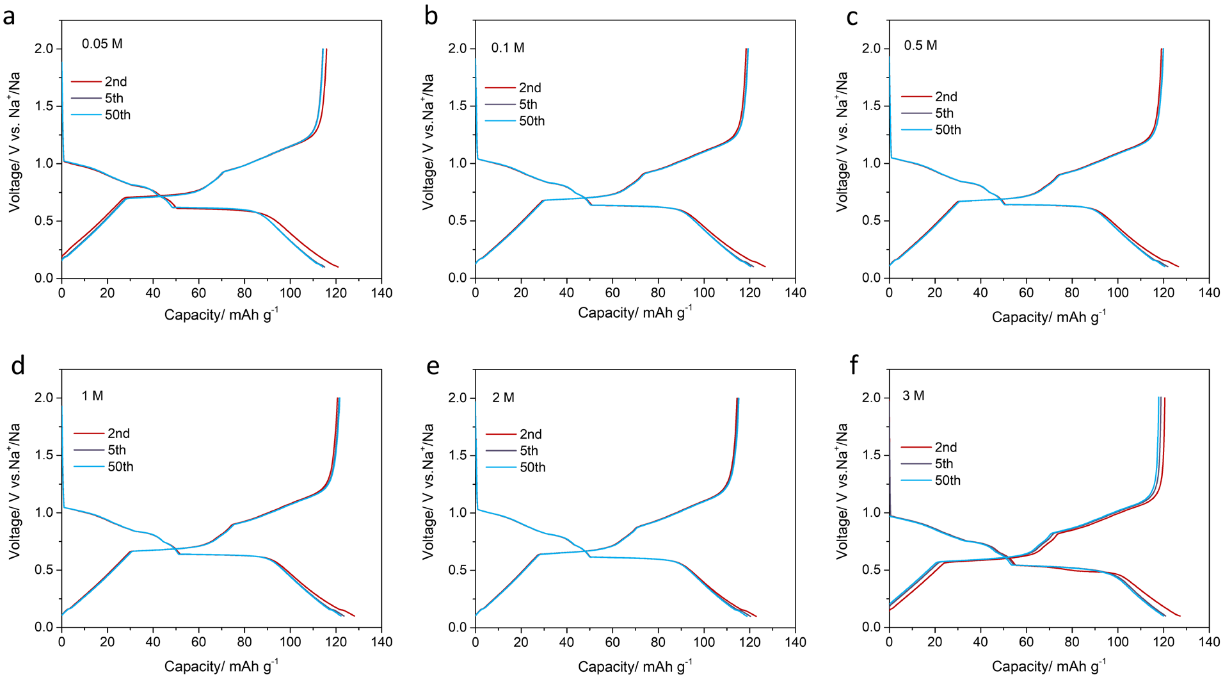

Supplementary Figure 3 | Voltage profiles of graphite anodes cycled in $NaPF_6$ G_2 electrolytes with different concentrations. (a) 0.05 M, (b) 0.1 M, (c) 0.5 M, (d) 1 M, (e) 2 M and (f) 3 M. The current density is 50 mA g^{-1}.

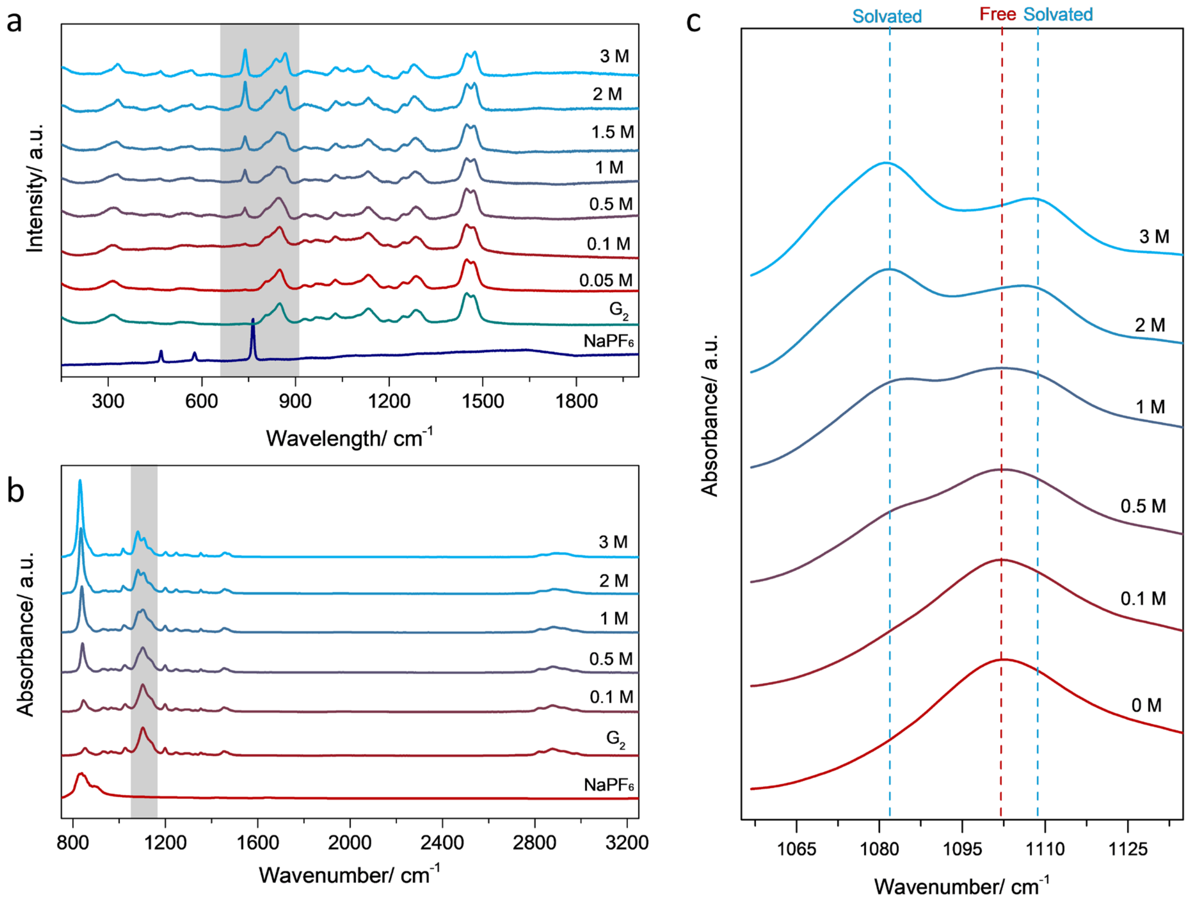

Supplementary Figure 4 | Raman and FTIR spectra of $NaPF_6$ G_2 electrolytes with increasing concentrations. (a) General Raman spectra, (b) general FTIR spectra and (c) selected FTIR spectra of $NaPF_6$ salt, G_2 solvent and $NaPF_6$ /G_2 electrolytes with different concentrations. Gray areas in (a) and (b) refers to selected area for further analyses.

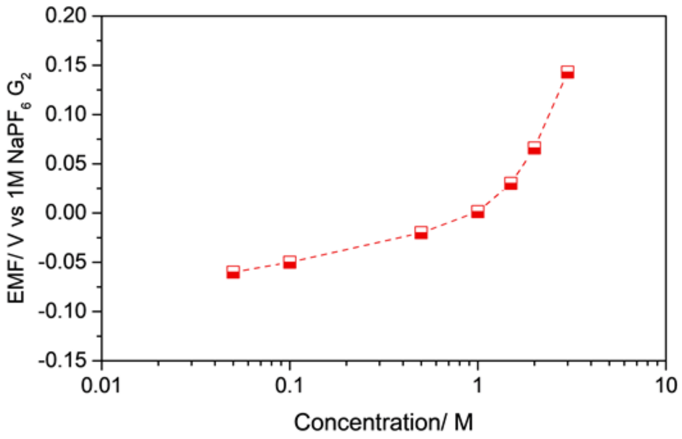

Supplementary Figure 5 | Plot of the EMF against the sodium salt concentration in G_2.

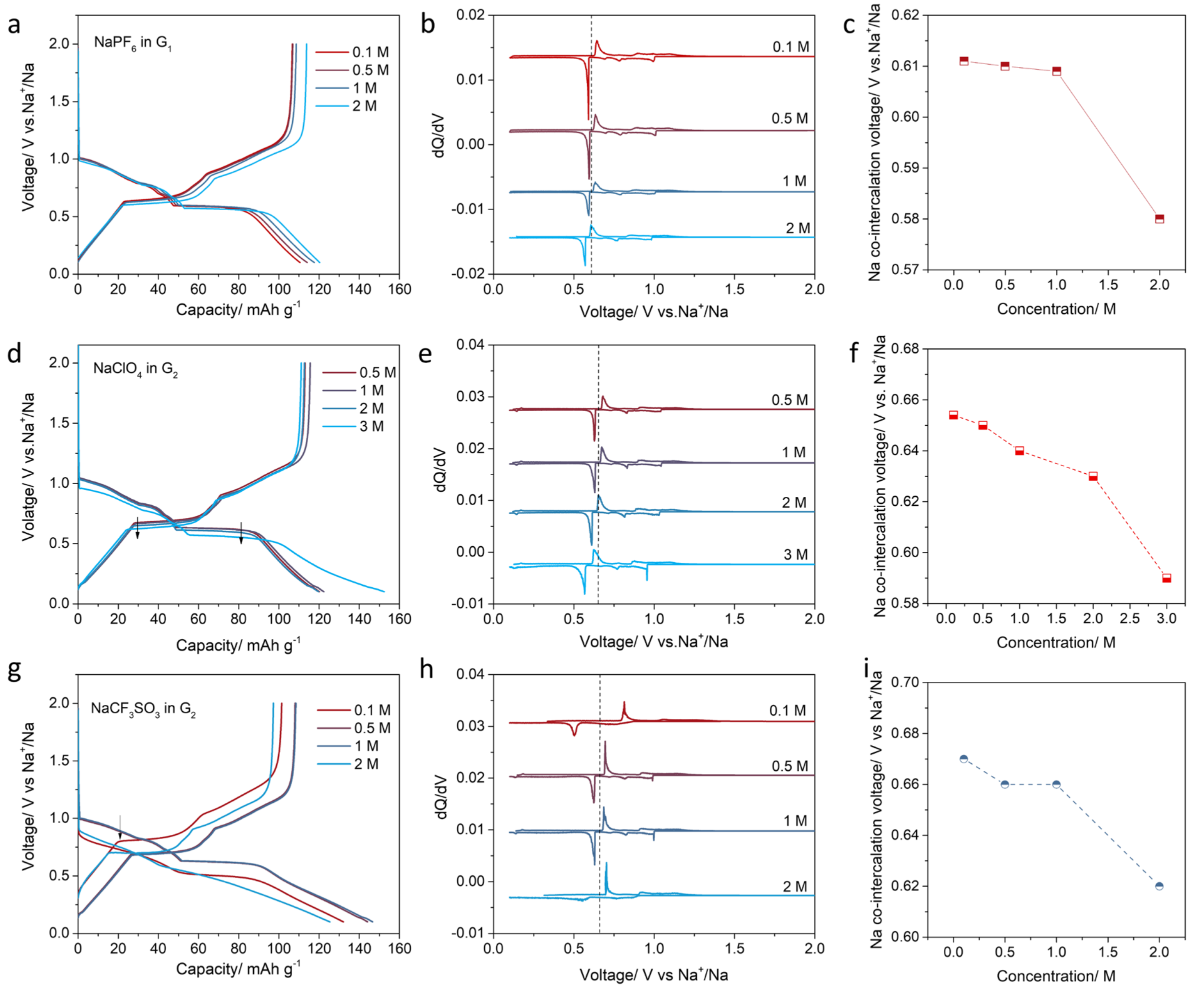

Supplementary Figure 6 | Downshift of co-intercalation voltage for graphite cycled in concentrated electrolytes regardless of salts and ether solvents. Galvanostatic charge/discharge profiles, the corresponding dQ/dV curves and the plots of average co-intercalation voltage in graphite as a function of electrolyte concentrations for (a-c) $NaPF_6$ dissolved in G_1 from 0.1 M to 2 M, (d-f) $NaClO_4$ dissolved in G_2 from 0.5 M to 3 M and (g-i) $NaCF_3SO_3$ dissolved in G_2 from 0.1 M to 2 M, respectively.

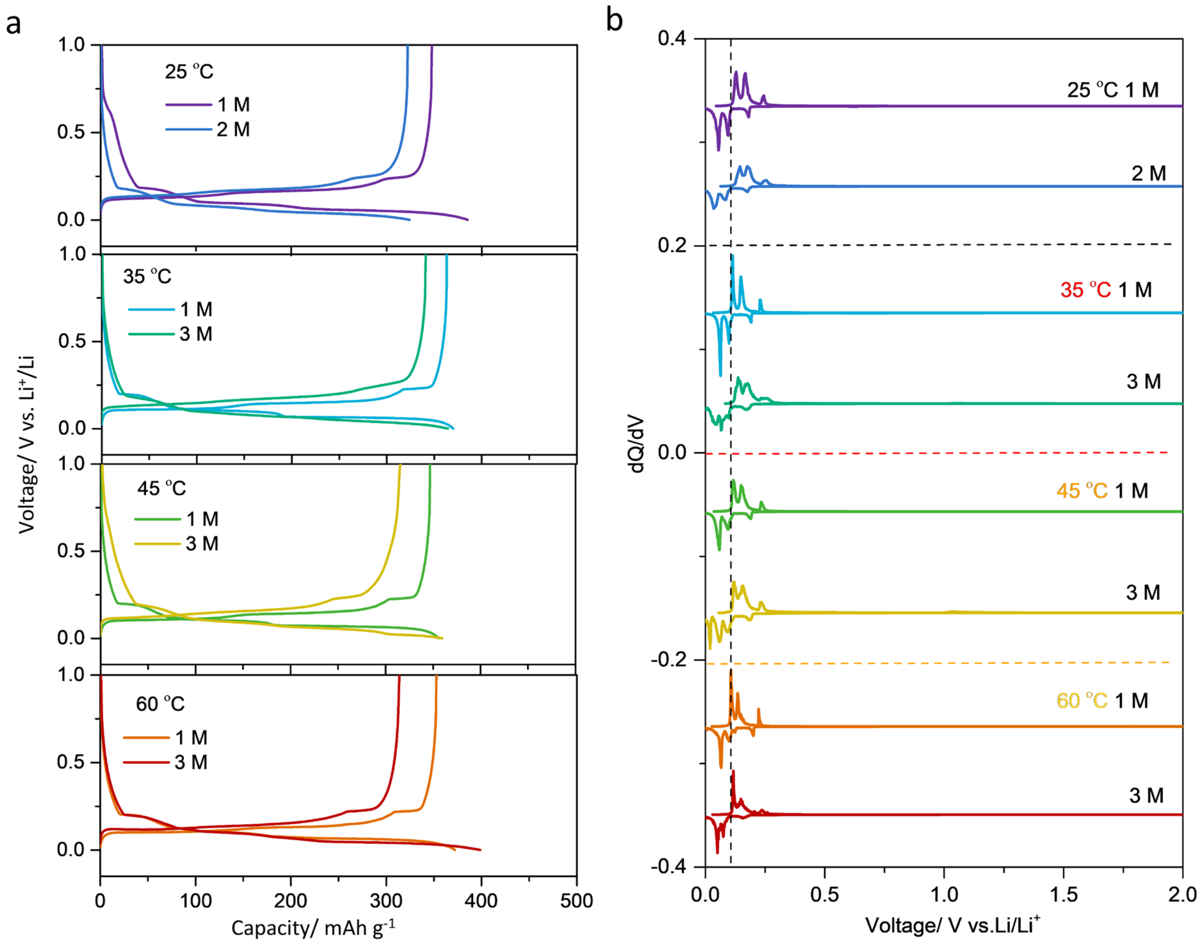

Supplementary Figure 7 | No shift of intercalation potential for graphite in Li-ion batteries. (a) Discharge/charge profiles of graphite anodes cycled in 1 M and 3 M $LiPF_6$ EC/DEC electrolytes at different temperatures of 25 °C, 35 °C, 45 °C and 60 °C, respectively, (b) the corresponding dQ/dV curves from (a) to show the average Li intercalation potential in graphite anodes under different conditions. Note that we used 2 M electrolyte for the comparison at 25 °C. That is because graphite presented very low reversible capacity and illegible dQ/dV peaks in 3 M electrolyte, possibly due to its high viscosity and poor electrode wettability. It reveals that the Li ion intercalation potentials in natural graphite are almost independent on the concentrations of electrolyte and operating temperatures.

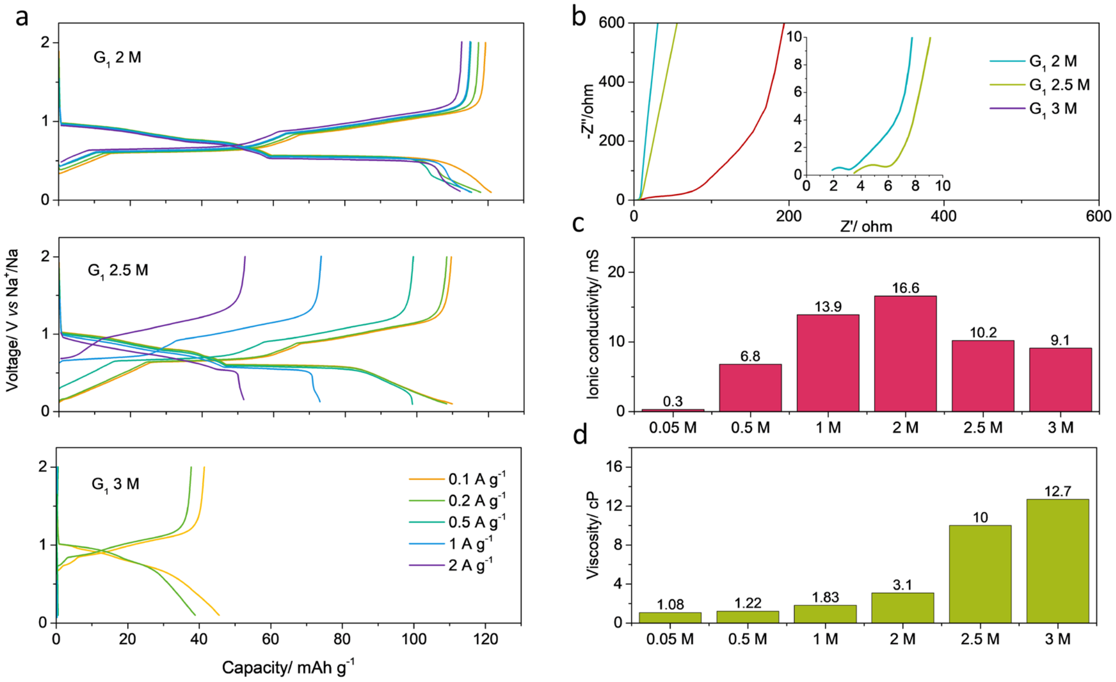

Supplementary Figure 8 | Rate performance of graphite and properties of concentrated G_1-based electrolytes and electrolyte properties. (a) Discharge/charge profiles of graphite electrodes in 2 M, 2.5 M and 3 M G_1-based electrolytes at 0.1, 0.2, 0.5, 1 and 2 A g^{-1}, (b) Nyquist plots of cycled electrodes in (a), (c) ionic conductivity and (d) viscosity of G_1-based electrolytes with concentrations ranging from 0.05 M to 3 M. According to the discussion in main content, higher electrolyte concentrations lead to lower co-intercalation potentials. To explore the limit of the benefits afforded from highly concentrated G_1-based electrolyte, we prepared electrolytes to 3 M, which is the saturation concentration at room temperature. In (a), it shows that the rate capacities decrease dramatically by using 2.5 M and 3 M G_1-based electrolytes. The poor rate performance is attributed to the (b) high charge transfer resistance, (c) the largely decreased ionic conductivity and (d) the significantly increased viscosity for 2.5 M and 3 M G_1-based electrolytes. In contrast, 2 M G_1-based electrolyte possesses the highest ionic conductivity and a moderate viscosity. Therefore, 2 M G_1-based electrolyte is considered close to the sweet spot in this work.

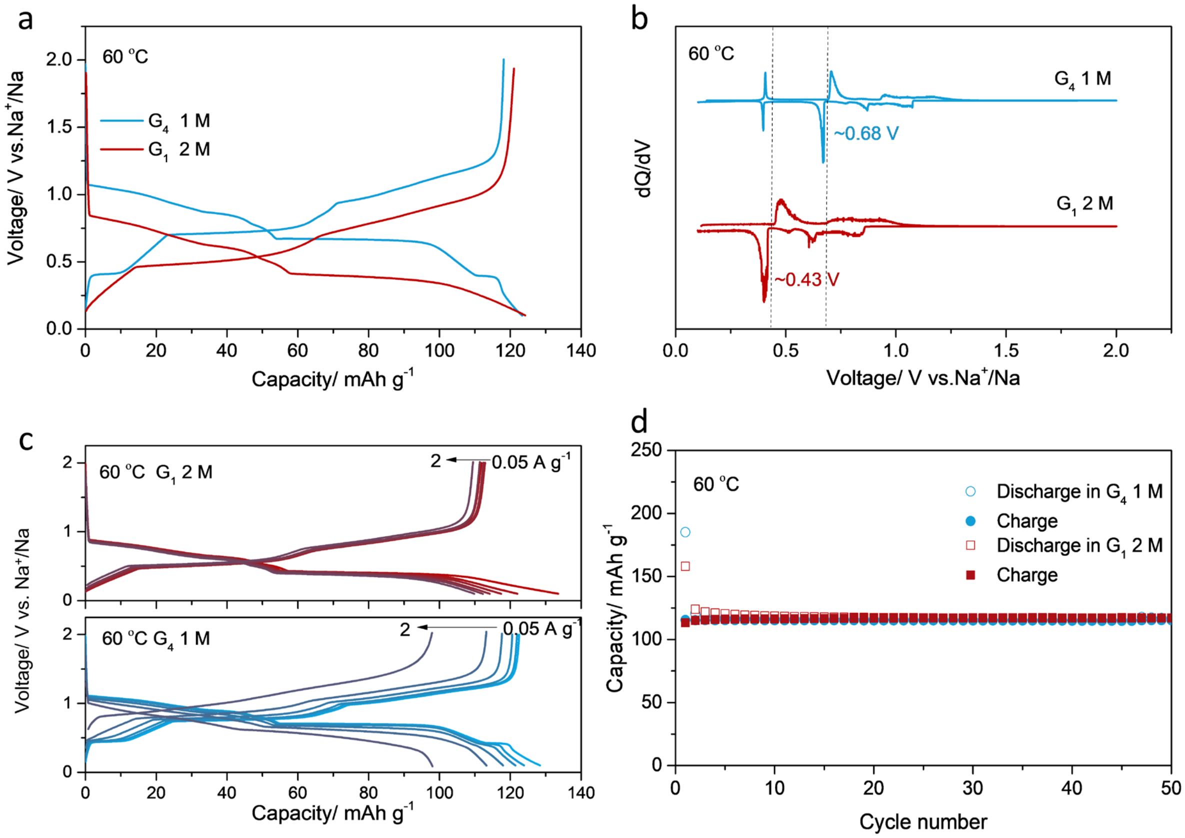

Supplementary Figure 9 | Comparison of electrochemical properties for graphite anodes cycled at 60 °C in 1 M $NaPF_6$ G_4 and 2 M $NaPF_6$ G_1 electrolytes. (a) Voltage profiles of the second charge/discharge processes, (b) the corresponding dQ/dV curves to show the average Na co-intercalation potentials, (c) rate capacities at 0.05, 0.1, 0.2, 0.5, 1 and 2 A g^{-1} and (d) cyclic performance at 0.1 A g^{-1} for 50 cycles.

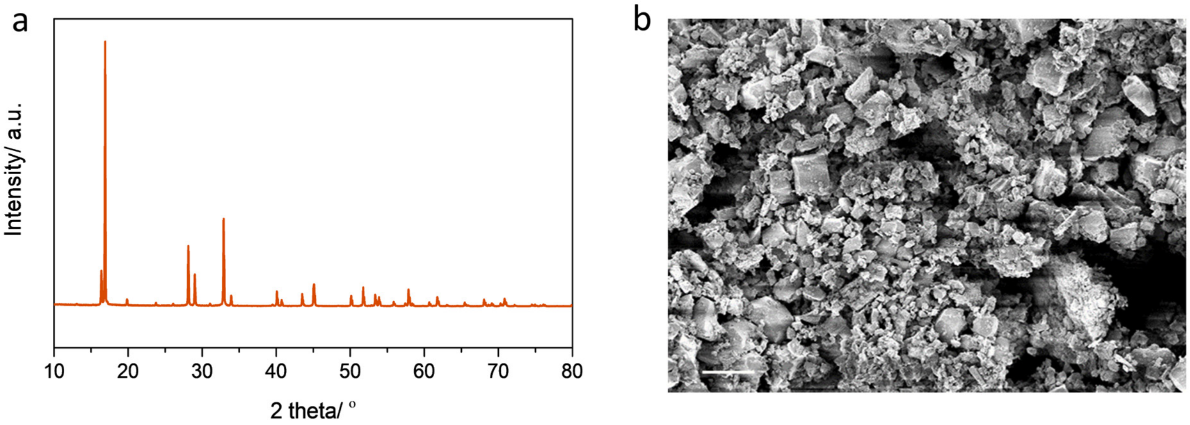

Supplementary Figure 10 | Structure of $Na_{1.5}VPO_{4.8}F_{0.7}$ cathode materials. (a) XRD pattern and (b) SEM image of $Na_{1.5}VPO_{4.8}F_{0.7}$ cathodes. The scale bar in (b) is 10 μm.

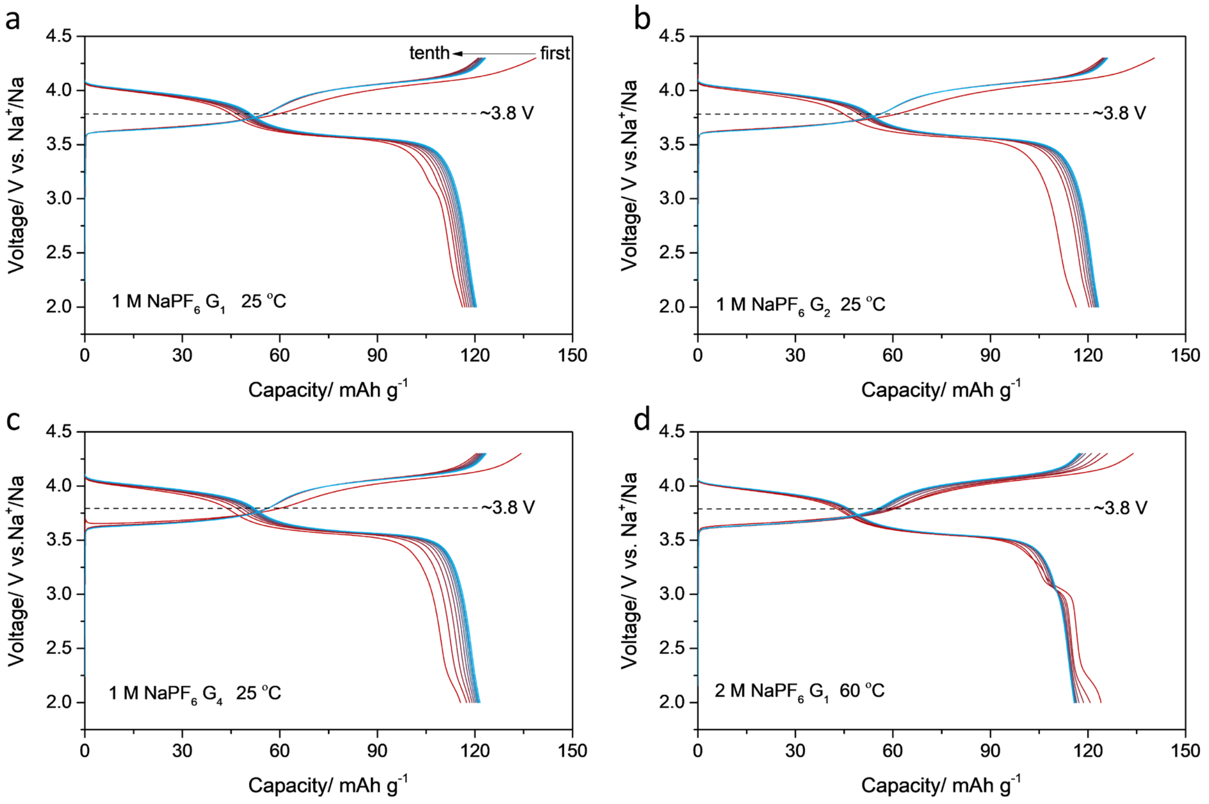

Supplementary Figure 11 | Cyclic performance of $Na_{1.5}VPO_{4.8}F_{0.7}$ cathodes under different conditions. Initial ten cycles charge/discharge profiles of $Na_{1.5}VPO_{4.8}F_{0.7}$ cathodes in (a) 1 M $NaPF_6$ G_1 electrolyte at 25 °C, (b) 1 M $NaPF_6$ G_2 electrolyte at 25 °C, (c) 1 M $NaPF_6$ G_4 electrolyte at 25 °C and (d) 2 M $NaPF_6$ G_1 electrolyte at 60 °C at a current density of 0.1 A g^{-1}.

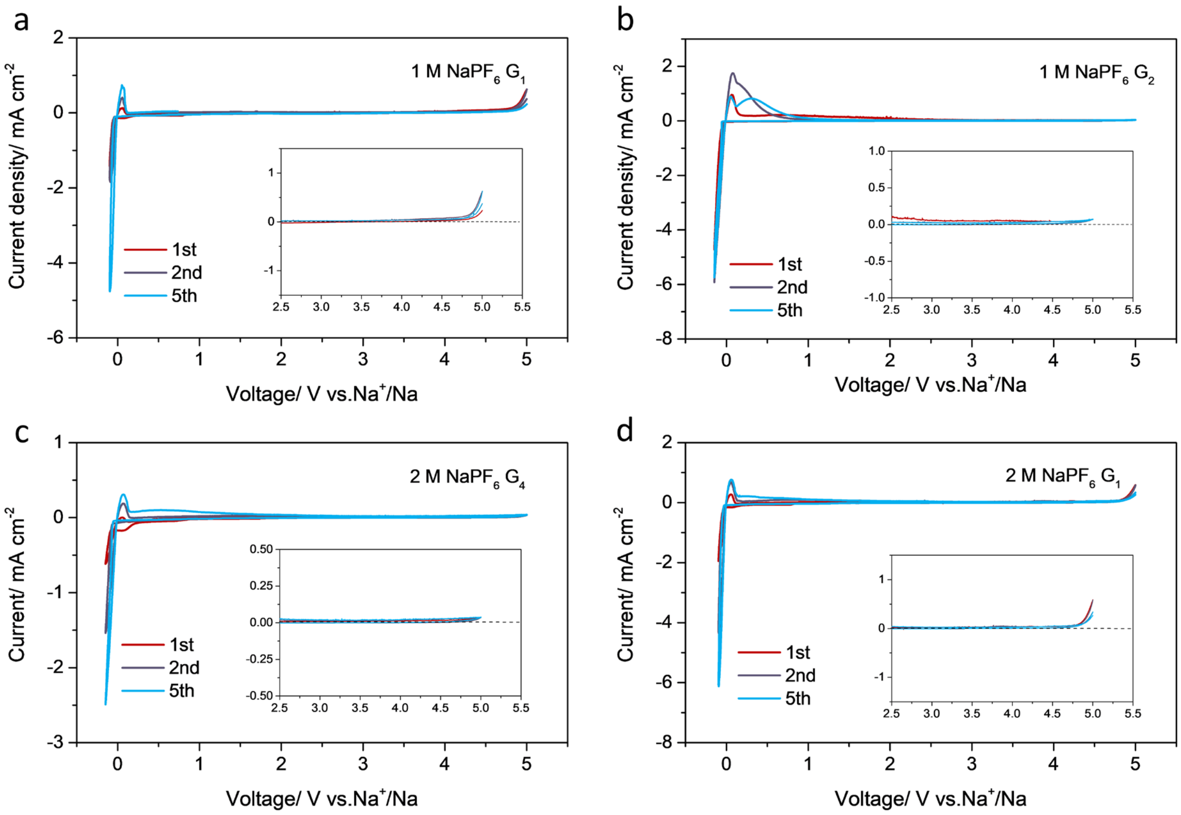

Supplementary Figure 12 | Electrochemical stability of electrolytes used for Na-ion full cells in this work. (a) 1 M $NaPF_6$ G_1, (b) 1 M $NaPF_6$ G_2, (c) 1 M $NaPF_6$ G_4 and (d) 2 M $NaPF_6$ G_1. To study the stable electrochemical windows of the electrolytes in this work, coin cells were assembled using a sodium foil as anode, a titanium foil as cathode, and different electrolytes. The area of titanium foil disc is 0.71 cm^{-2}. The coin cells were subjected to a CV test at a scan rate of 2 mV s^{-1} between -0.25 V and 5.0 V.

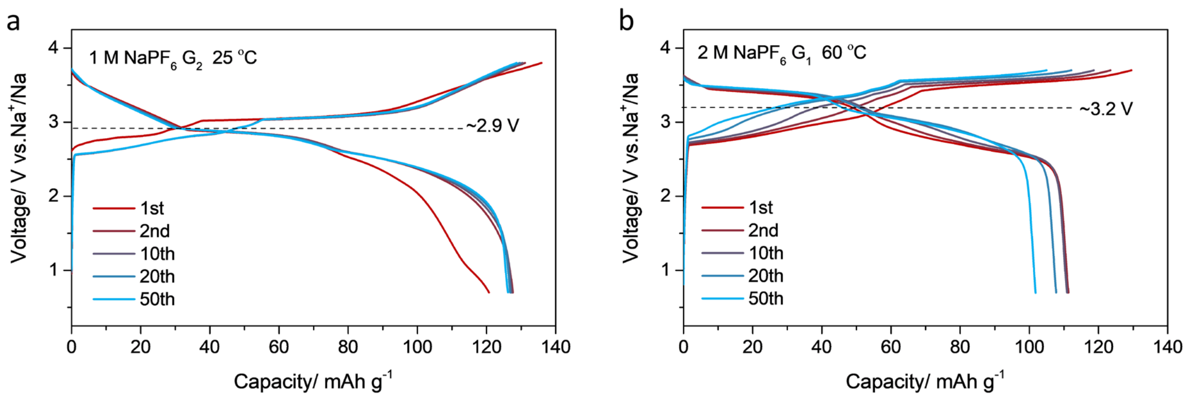

Supplementary Figure 13 | Discharge/charge profiles of graphite|| $Na_{1.5}VPO_{4.8}F_{0.7}$ full cells under other measurement conditions. (a) 1 M $NaPF_6$ G_2 electrolyte at 25 °C and (b) 2 M $NaPF_6$ G_1 electrolyte at 60 °C. The Na ion full cells were cycled at 0.1 A g^{-1} for 50 cycles, which present average output voltage of 2.9 V and 3.2 V in (a) and (b), respectively. The cyclic capacity degradation in (b) may be attributed to electrolyte decomposition at high temperature.

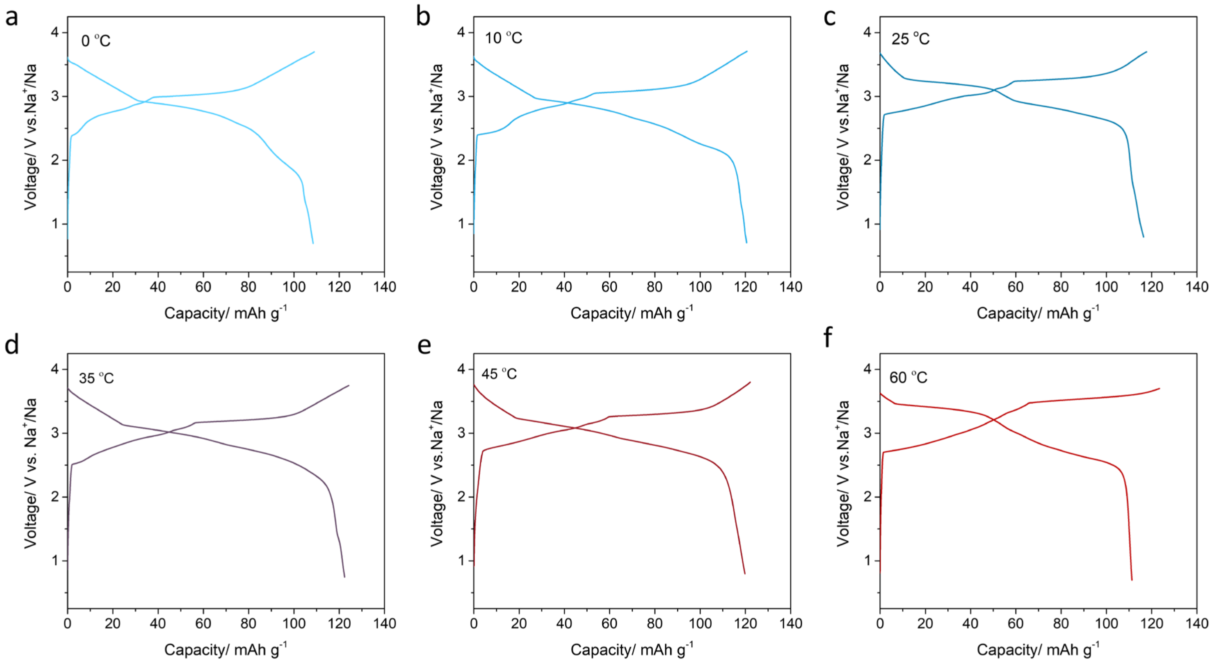

Supplementary Figure 14 | Charge/discharge profiles of graphite|| $Na_{1.5}VPO_{4.8}F_{0.7}$ full cells cycled under different temperatures. (a) 0 °C, (b) 10 °C, (c) 25 °C, (d) 35 °C, (e) 45 °C and (f) 60 °C in 2 M G_1-based electrolyte at 0.1 A g^{-1}.

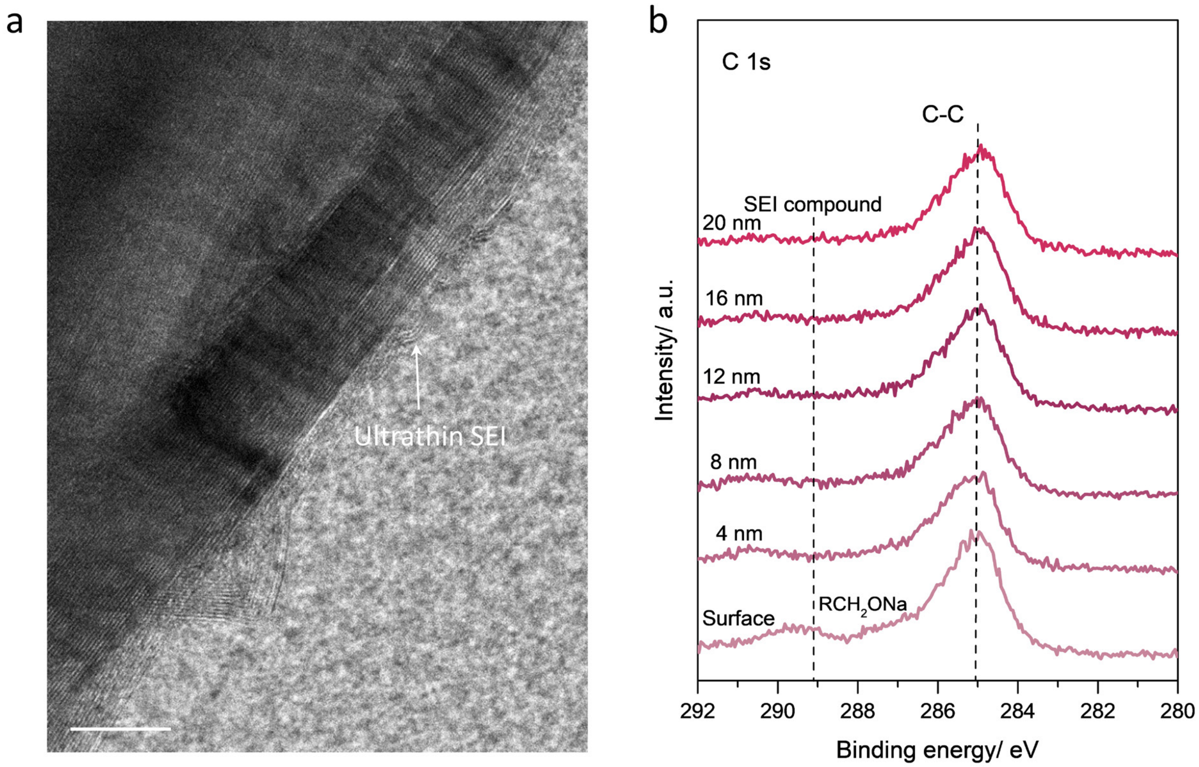

Supplementary Figure 15 | Characterization of SEI layer on graphite electrodes after 5 cycles in 2 M $NaPF_6$ G_1 electrolyte. (a) TEM image, (b) XPS spectra. (a) indicates that no noticeable SEI layer was formed at the surface of graphite. The XPS results in (b) with depth profiling (from surface to 20 nm) also clearly show that the amount of SEI on cycled graphite is rather trivial. The scale bar in (a) is 10 nm.

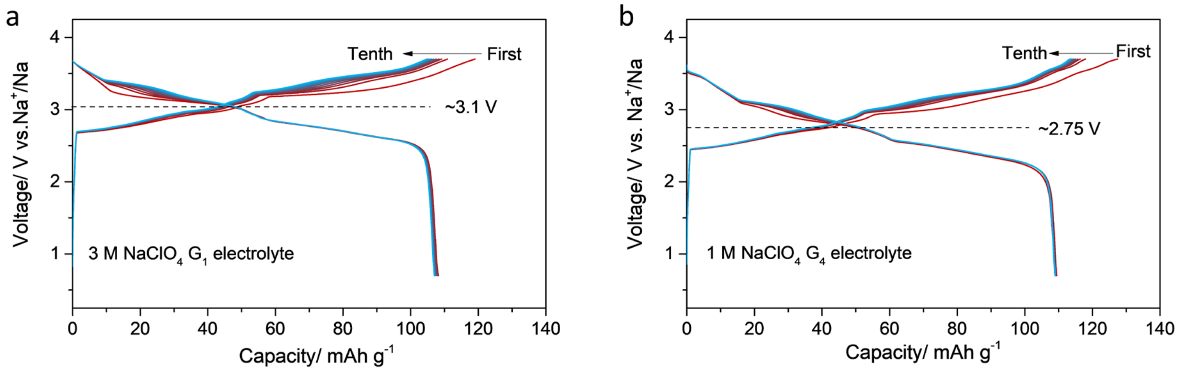

Supplementary Figure 16 | First ten cycles' discharge/charge profiles of graphite|| $Na_{1.5}VPO_{4.8}F_{0.7}$ full cells in $NaClO_4$-based electrolytes. (a) 3 M $NaClO_4$ G_1 electrolyte and (b) 1 M $NaClO_4$ G_4 electrolyte. The performance of Na-ion full cells in $NaClO_4$-based electrolyte is very similar with that in $NaPF_6$-based electrolyte, in terms of output voltage and cyclic capacities. Considering that the price of $NaClO_4$ salt ($50 per 100g, supplied by Aldrich) is much cheaper than $NaPF_6$ ($186 per 50g, supplied by Aldrich), $NaClO_4$-based highly concentrated electrolyte is promising to lower the cost for high energy graphite||$Na_{1.5}VPO_{4.8}F_{0.7}$ full cells.

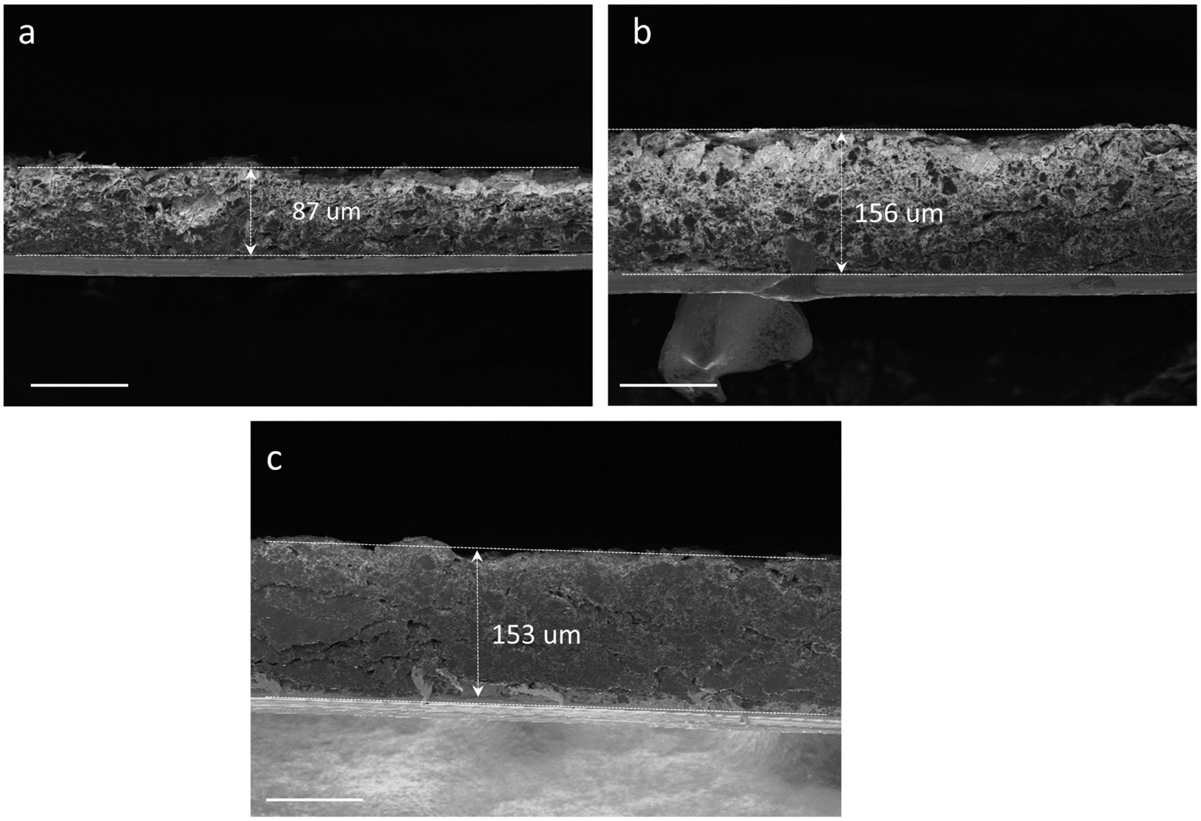

Supplementary Figure 17 | Morphological characterization of fully discharged graphite electrodes. SEM images of (a) pristine, (b) 1st discharged, and (c) 10th discharged graphite electrodes. The volume expansion is calculated to be about 80 % and remains stable during cycles. The scale bars in (a-c) are 100 μm.

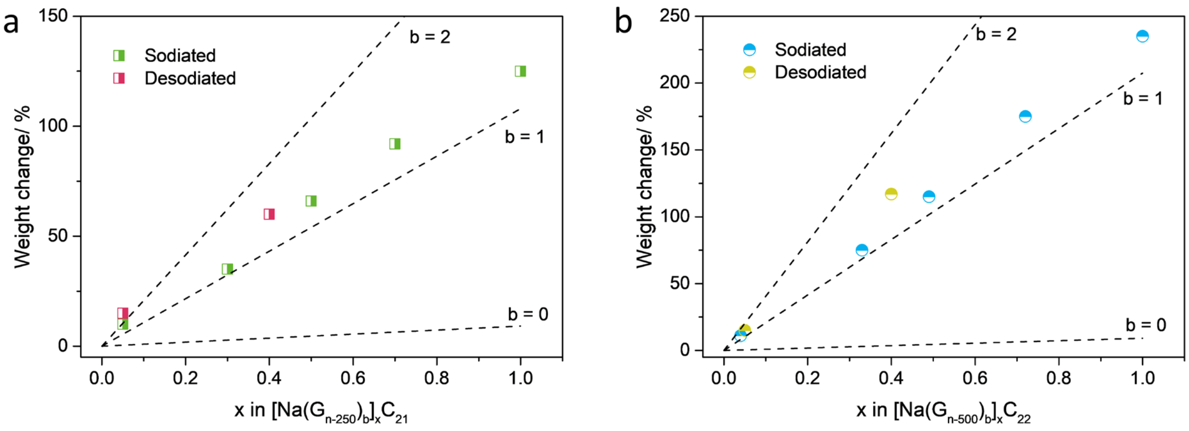

Supplementary Figure 18 | Determination of the coordination number of Na ions with $G_{n\text{-}250}$ and $G_{n\text{-}500}$ molecules. Weight change of graphite measured in various states of sodiation and desodiation to determine the *b* values in (a) $[Na(G_{n\text{-}250})_b]_xC_{21}$ and (b) $[Na(G_{n\text{-}500})_b]_xC_{22}$. In both cases, *b* is near to be 1, suggesting one Na ion is solvated with one $G_{n\text{-}250}$ or $G_{n\text{-}500}$ molecule in co-intercalated graphite. It is worth noting that $G_{n\text{-}250}$ and $G_{n\text{-}500}$ are mixture of long chain ethers (*i.e.,* G_n with average n = 4.6 and 10.3, respectively). Although they are not pure ether molecules, the co-intercalation voltages of graphite in $G_{n\text{-}250}$ and $G_{n\text{-}500}$-based electrolytes also followed the trend that the average co-intercalation voltage increases with the average chain length of the solvent. In addition, one $G_{n\text{-}250}$ or $G_{n\text{-}500}$ ether molecule tends to coordinate with one Na ion in the electrolyte according to previous studies.[1,2]

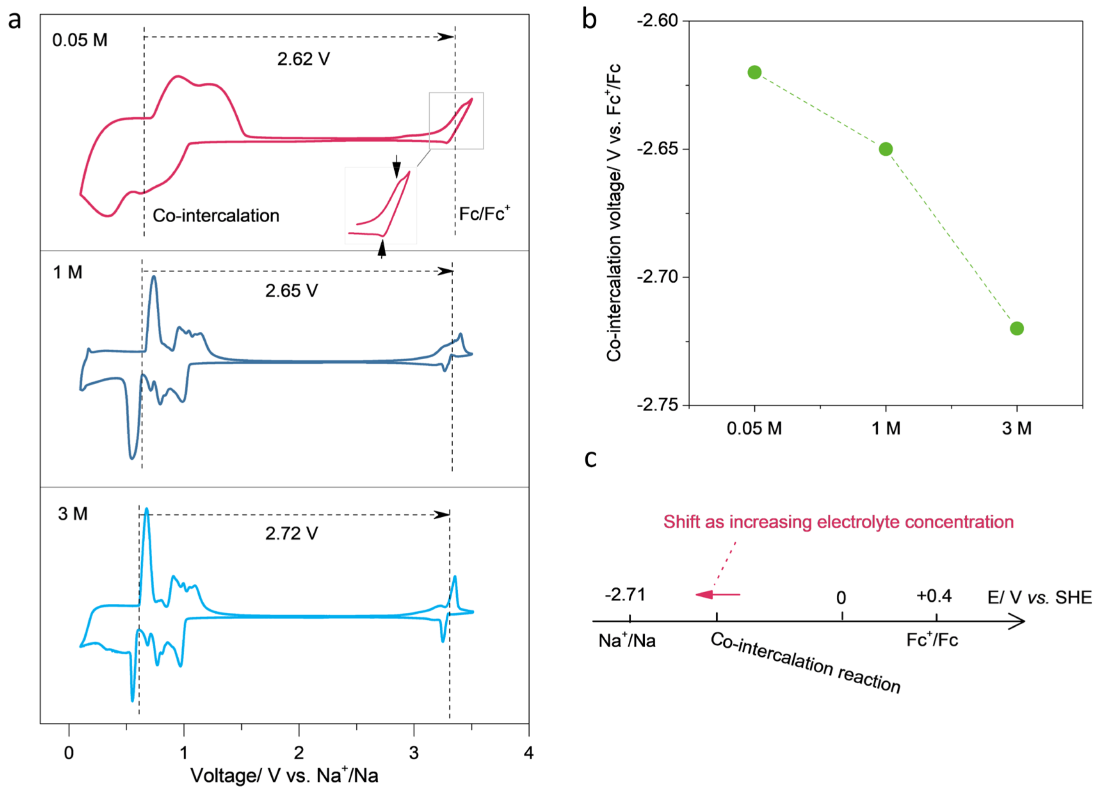

Supplementary Figure 19 | Confirmation of the shift of co-intercalation potential by using ferrocene internal reference. (a) Cyclic voltammetry curves of graphite electrode cycled in 0.05 M, 1 M and 3 M $NaPF_6$ G_2 electrolytes containing 10 mM ferrocene. The experiments were conducted in three electrode systems using graphite cast on Ni mesh as working electrodes, Pt wire as counter electrodes and Na metal as reference electrodes[3]. The scan rate is 1 mV s^{-1}. (b) the average co-intercalation voltages *vs*. Fc^+/Fc as a function of electrolyte concentrations, (c) schematically showing the left-shift of co-intercalation potential versus Na^+/Na and Fc^+/Fc references. Considering Fc^+/Fc the best known internal reference[4], the average co-intercalation voltages *vs*. Fc^+/Fc are regarded negative shift as increasing electrolyte concentrations, confirming our finding about the concentration dependent shift of the co-intercalation voltage. Note that the Fc^+/Fc redox couple in 0.05 M electrolyte is enlarged to index due to the large polarization in the extremely dilute electrolyte.

Supplementary Table 1 | Bader charge of $[Na\text{-}G_n]^+$ complexes co-intercalated t-GICs. Regardless of the solvent species, amount of charge transfer is almost identical.

$[Na\text{-}G_n]^+$ complex	$[Na\text{-}G_1]^+$	$[Na\text{-}G_2]^+$	$[Na\text{-}G_4]^+$
Bader charge	+0.89	+0.89	+0.90

Supplementary Table 2 | Comparison of the recently reported Na ion full cell configurations with the graphite||$Na_{1.5}VPO_{4.8}F_{0.7}$ full cells in this work.

Full cell (anode||cathode)	Voltage /V	Cyclic performance (initial reversible capacity, cycle number, capacity retention)	Energy and power densities	Ref.
$Na_2Ti_3O_7$||$VOPO_4$	2.9	113.9 mAh g^{-1}, 100, 92.3%,	220 Wh kg^{-1} at ~22 W kg^{-1}, 100 Wh kg^{-1} at 1514 W kg^{-1}	5
[c]Fe_3O_4|| $Na[Ni_{0.25}Fe_{0.5}Mn_{0.25}]O_2$	2.4	130 mAh g^{-1}, 150, 76 %	312 Wh kg^{-1} at 31.2 W kg^{-1}, 172 Wh kg^{-1} at 1720 W kg^{-1}	6
Fe_3O_4||$Na_2FeP_2O_7$	2.28	93 mAh g^{-1}, 100, 93.3%	130 Wh kg^{-1} at ~13 W kg^{-1}, 75 Wh kg^{-1} at 1600 W kg^{-1}	7
$Na_{0.6}[Cr_{0.6}Ti_{0.4}]O_2$ symmetric	2.5	80 mAh g^{-1}, 100, 81 %	94 Wh kg^{-1} at 18.8 W kg^{-1}, 82 Wh kg^{-1} at 82 W kg^{-1}	8
Sn||graphite dual ion battery	4.25	~40 mAh g^{-1}, 50, ~92 %	144 Wh kg^{-1} at 150 W kg^{-1}, 111 Wh kg^{-1} at 793 W kg^{-1}	9
Sb@TiO_{2-x}|| $Na_3V_2(PO_4)_3$	1.8-2.1	~350 mAh g^{-1}, 100, ~63 %	151 Wh kg^{-1} at 21 W kg^{-1}, 61 Wh kg^{-1} at 1830 W kg^{-1}	10
$NaTi_2(PO_4)_3$|| $Na_{0.44}MnO_2$	~1.2	~120 mAh g^{-1}, 100, ~65 %	30 Wh kg^{-1} at ~80 W kg^{-1}, 2.5 Wh kg^{-1} at ~2800 W kg^{-1}	11
Hard carbon|| $Na_{0.9}[Cu_{0.22}Fe_{0.3}Mn_{0.48}]O_2$	~3.2	300 mAh g^{-1}, 100, ~100 %	210 Wh kg^{-1} at 105 W kg^{-1}, ~136 Wh kg^{-1} at ~815 W kg^{-1}	12
Hard carbon|| $NaNi_{1/3}Fe_{1/3}Mn_{1/3}O_2$	~2.9	1Ah soft battery, 500, 73 %	/	13
Graphite||$Na_{0.7}CoO_2$	2.2	80 mAh g^{-1}, 100, ~94 %	60 Wh kg^{-1} at 60 W kg^{-1}, 30 Wh kg^{-1} at 300 W kg^{-1}	14
Porous graphite|| $Na_3V_2(PO_4)_3$	~2.7	109 mAh g^{-1}, 500, 92 %	131 Wh kg^{-1} at ~24 W kg^{-1}, ~85 Wh kg^{-1} at 392 W kg^{-1}	15
Graphite|| $Na_3V_2(PO_4)_3$	2.2	92 mAh g^{-1}, 400, 80 %	76.2 Wh kg^{-1} at ~76 W kg^{-1}, ~65 Wh kg^{-1} at ~1444 W kg^{-1}	16
Graphite|| $Na_{1.5}VPO_{4.8}F_{0.7}$	3.1	108 mAh g^{-1}, 1000, 93 %	149 Wh kg^{-1} at 125 W kg^{-1}, 112 Wh kg^{-1} at 3863 W kg^{-1}	This work

[c] The specific capacity, energy and power densities of this full cell are calculated based on the mass of cathode materials. While, for other full cells, the specific capacities are calculated based on the mass of anode materials, the energy and power densities are calculated based on the total mass of anode and cathode materials.

Supplementary Note 1 | Discussion of electrode potentials

While shifts in the co-intercalation voltage caused by the change of electrolyte concentration are thoroughly depicted in the main context, and were confirmed by using ferrocene as the internal reference (Supplementary Fig. 19), clear explanations as to why the plateau voltage changes in Na||graphite half-cell at highly concentrated electrolyte is provided here.

For a Na metal|| 1M $NaPF_6$ G_2|| graphite half-cell, the electrode reactions can be described as:

Anode: $Na + (G_2) \leftrightarrow [Na\text{-}G_2]^+ + e^-$ (1)

Cathode: $xC + [Na\text{-}G_2]^+ + e^- \leftrightarrow [Na\text{-}G_2]C_x$ (2)

Overall: $Na + xC + (G_2) \leftrightarrow [Na\text{-}G_2]C_x$ (3)

Accordingly, the electrode potentials can be derived from Nernst Equations:

Anode: $E_{Na} = E^0_{Na} + \frac{2.303RT}{F}\log\frac{a_{[Na-G2]^+}}{a_{G2}}$ (4)

Cathode: $E_{t\text{-}GIC} = E^0_{t-GIC} + \frac{2.303RT}{F}\log a_{[Na-G2]^+}$ (5)

Overall voltage: $V = E_{t\text{-}GIC} - E_{Na} = (E^0_{t-GIC} - E^0_{Na}) + \frac{2.303RT}{F}\log a_{G2}$ (6)

Where the E^0_{Na} is the standard electrode potential for the equilibrium, E^0_{t-GIC} is the standard electrode potential for $[Na\text{-}G_2]^+$ co-intercalation reaction, F is the Faraday constant, R is the gas constant, T is the absolute temperature and $a_{[Na-G2]^+}$ and a_{G2} are activities of $[Na\text{-}G_2]^+$ and free G_2, respectively. When the electrolyte concentration increases from 1 M to 3 M, the Na electrode potential change can be described as:

$\Delta E_{Na} = \frac{2.303RT}{F}(\log\frac{a_{[Na-G2]^+,3M}}{a_{G2,3M}} - \log\frac{a_{[Na-G2]^+,1M}}{a_{G2,1M}}) = \frac{2.303RT}{F}(\log\frac{a_{[Na-G2]^+,3M}}{a_{[Na-G2]^+,1M}} + \log\frac{a_{G2,1M}}{a_{G2,3M}})$ (7)

the co-intercalation potential change can be described as:

$\Delta E_{t\text{-}GIC} = \frac{2.303RT}{F}\log\frac{a_{[Na-G2]^+,3M}}{a_{[Na-G2]^+,1M}}$ (8)

Where the $a_{[Na-G2]^+,3M}$, $a_{[Na-G2]^+,1M}$, $a_{G2,3M}$ and $a_{G2,1M}$ refer to the activities of $[Na\text{-}G_2]^+$ and free G_2 at 3 M and 1 M electrolyte, respectively. It is observed that the potential of Na metal electrode increases with the increase of $[Na\text{-}G_2]^+$ activity and the decrease of free G_2 activity in highly concentrated electrolyte. The potential of graphite electrode also increases by $\frac{2.303RT}{F}\log\frac{a_{[Na-G2]^+,3M}}{a_{[Na-G2]^+,1M}}$ when the concentration of electrolyte increases from 1 M to 3 M. The overall co-intercalation voltage change is determined by the activity change of free G_2 molecules (*i.e.*, $\Delta V=\frac{2.303RT}{F}\log\frac{a_{G2,3M}}{a_{G2,1M}}$). Since the $a_{G2,3M}$ at highly concentrated electrolyte is smaller than $a_{G2,1M}$, ΔV should be a negative value, corresponding to the negative shift of co-intercalation voltage by increasing the electrolyte concentration. The thermodynamic analyses are in good agreement with the experimental results. It can be demonstrated that the decreasing solvent activity at high concentration induces the negative shift of co-intercalation voltages, which is an intrinsic property for co-intercalation reactions.

Supplementary Note 2 | Energy density of optimal graphite||$Na_{1.5}VPO_{4.8}F_{0.7}$ full cells

The optimal full cells in this work use 2 M $NaPF_6$ G_1 electrolyte, graphite anodes and $Na_{1.5}VPO_{4.8}F_{0.7}$ cathodes. The sodium storage capacity in graphite is 120 mAh g^{-1}, leading to a $[Na\text{-}G_1]C_{19}$ ternary graphite intercalation compound (t-GIC). Based on our previous work, the molar ratio of Na ion: G_1 of intercalated solvated-Na-ion is 1[17]. The Na ions and G_1 molecules are provided by $Na_{1.5}VPO_{4.8}F_{0.7}$ cathode and electrolyte, respectively. Thus, when we assume that 1 mol graphite is fully co-intercalated with the solvent, the theoretical content of G_1 molecules from electrolyte should be no less than 1/19 mol. Then, the least amount of electrolyte is calculated to be 6.58 g (in 2 M $NaPF_6$ G_1 electrolyte, the molar ratio of $NaPF_6$: G_1 is 2: 9.63; thus the mass of electrolyte can be calculated as $\frac{1}{19}mol \times 90.12\frac{g}{mol} + \frac{1}{19}mol \times \frac{2}{9.63} \times 167.9\frac{g}{mol} = 6.58g$). The ultimate electrolyte/graphite electrode mass ratio is evaluated to be 0.55 (or 0.22 for electrolyte/electrodes with cathode: anode mass ratio =1.5 in this work). Given the theoretical capacities of about 120 mAh g^{-1} for graphite anode (Fig. 3g) and $Na_{1.5}VPO_{4.8}F_{0.7}$ cathode (Fig. 4a) and an output voltage of 3.1 V, the theoretical energy density of a graphite||$Na_{1.5}VPO_{4.8}F_{0.7}$ full cell is calculated to be about 146 Wh kg^{-1} based on the total mass of anode, cathode and the least amount of electrolyte. In this work, we used excess amount of electrolyte (*i.e.*, 100 µl, mass density of about 1 mg μl^{-1}) to estimate the cyclic stability of graphite||$Na_{1.5}VPO_{4.8}F_{0.7}$ full cells. The electrolyte/electrode mass ratio is calculated to be 5.26, considering the anode: cathode mass ratio = 1.5 and graphite mass loading of 6 mg cm^{-2} (electrode diameter = ½ inch). Accordingly, the practical energy density is estimated to be only 23.8 Wh kg^{-1}, which is far from the theoretical value. Apparently, more efforts are needed to decrease the electrolyte/electrode ratio to approach the high theoretical energy density.

Supplementary Methods

In order to obtain the repulsion energy from negatively charged graphene layers, we performed density functional theory calculations using Vienna Ab initio Simulation Package (VASP) program.[18] A projector-augmented wave pseudopotential with a plane-wave basis sets was used as implemented in VASP.[19,20] Exchange-correlation energies were treated with generalized gradient energy of Perdew-Burke-Ernzerhof.[21] The consideration of Van der Waals force between graphene layers was included by DFT-D3 functional.[22] Energy cutoff was set to 500 eV and the unit cell was converged until the energy difference became less than 0.01 eV during iterations for initial optimization of graphite.

The graphite structure used for the repulsion energy calculation contains 48 carbon atoms in a unit cell, and two electrons were additionally injected to the unit cell to describe the negatively charged graphene layers in co-intercalated states. This amount of charge injection reflects the charged state of carbon layers in [Na-ether]C_{20}, which shows theoretical capacity of 110 mAh g^{-1}, close to the reversible capacity observed in our experiments. Then we expanded the interlayer distance between graphene layers and obtained the free energy of each system to get the repulsion energy between charged graphene layers as a function of interlayer distance. In order to obtain the amount of charge transferred from intercalants to graphene layers, Bader charge analysis was conducted for G_1, G_2 and G_4 co-intercalated graphite.[23]

Supplementary References

1. Chan, L. L. & Smid, J. Contact and solvent-separated ion pairs of carbanions. IV. Specific solvation of alkali ions by polyglycol dimethyl ethers. *J. Am. Chem. Soc.* **89,** 4547–4549 (1967).
2. Chan, L. L., Wong, K. H. & Smid, J. Complexation of lithium, sodium, and potassium carbanion pairs with polyglycol dimethyl ethers (glymes). Effect of chain length and temperature. *J. Am. Chem. Soc.* **92,** 1955–1963 (1970).
3. Mozhzhukhina, N. & Calvo, E. J. Perspective—The correct assessment of standard potentials of reference electrodes in non-aqueous solution. *J. Electrochem. Soc.* **164,** A2295–A2297 (2017).
4. Gritzner, G. & Kuta, J. Recommendations on reporting electrode potentials in nonaqueous solvents. *Pure Appl. Chem.* **56,** 464–466 (1984).
5. Li, H. *et al.* An advanced high-energy sodium ion full battery based on nanostructured $Na_2Ti_3O_7/VOPO_4$ layered materials. *Energy Environ. Sci.* **9,** 3399–3405 (2016).
6. Oh, S. M. *et al.* Advanced $Na[Ni_{0.25}Fe_{0.5}Mn_{0.25}]O_2/C–Fe_3O_4$ sodium-ion batteries using EMS electrolyte for energy storage. *Nano Lett.* **14,** 1620–1626 (2014).
7. Ming, J. *et al.* A sustainable iron-based sodium ion battery of porous carbon–$Fe_3O_4/Na_2FeP_2O_7$ with high performance. *RSC Adv.* **5,** 8793–8800 (2015).
8. Wang, Y., Xiao, R., Hu, Y. S., Avdeev, M. & Chen, L. P2-$Na_{0.6}[Cr_{0.6}Ti_{0.4}]O_2$ cation-disordered electrode for high-rate symmetric rechargeable sodium-ion batteries. *Nat. Commun.* **6,** 6954 (2015).
9. Sheng, M., Zhang, F., Ji, B., Tong, X. & Tang, Y. A novel Tin-graphite dual-ion battery based on sodium-ion electrolyte with high energy density. *Adv. Energy Mater.* **7,** 1601963

(2017).

10. Wang, N., Bai, Z., Qian, Y. & Yang, J. Double-walled Sb@TiO_{2-x} nanotubes as a superior high-rate and ultralong-lifespan anode material for Na-ion and Li-ion batteries. *Adv. Mater.* **28,** 4126–4133 (2016).

11. Li, Z., Young, D., Xiang, K., Carter, W. C. & Chiang, Y.-M. Towards high power high energy aqueous sodium-ion batteries: the $NaTi_2(PO_4)_3/Na_{0.44}MnO_2$ system. *Adv. Energy Mater.* **3,** 290–294 (2013).

12. Mu, L. *et al.* Prototype sodium-ion batteries using an air-stable and Co/Ni-free O3-layered metal oxide cathode. *Adv. Mater.* **27,** 6928–6933 (2015).

13. Wang, H. *et al.* Large-scale synthesis of $NaNi_{1/3}Fe_{1/3}Mn_{1/3}O_2$ as high performance cathode materials for sodium ion batteries. *J. Electrochem. Soc.* **163,** A565–A570 (2016).

14. Hasa, I. *et al.* A sodium-ion battery exploiting layered oxide cathode, graphite anode and glyme-based electrolyte. *J. Power Sources* **310,** 26–31 (2016).

15. Han, P. *et al.* Flexible graphite film with laser drilling pores as novel integrated anode free of metal current collector for sodium ion battery. *Electrochem. commun.* **61,** 84–88 (2015).

16. Zhu, Z., Cheng, F., Hu, Z., Niu, Z. & Chen, J. Highly stable and ultrafast electrode reaction of graphite for sodium ion batteries. *J. Power Sources* **293,** 626–634 (2015).

17. Kim, H. *et al.* Sodium intercalation chemistry in graphite. *Energy Environ. Sci.* **8,** 2963–2969 (2015).

18. Kresse, G. & Furthmüller, J. Efficiency of ab-initio total energy calculations for metals and semiconductors using a plane-wave basis set. *Comput. Mater. Sci.* **6,** 15–50 (1996).

19. Blöchl, P. E. Projector augmented-wave method. *Phys. Rev. B* **50,** 17953–17979 (1994).

20. Joubert, D. From ultrasoft pseudopotentials to the projector augmented-wave method. *Phys.*

Rev. B - Condens. Matter Mater. Phys. **59,** 1758–1775 (1999).

21. Perdew, J. P., Burke, K. & Ernzerhof, M. Generalized gradient approximation made simple. *Phys. Rev. Lett.* **77,** 3865–3868 (1996).

22. Grimme, S., Ehrlich, S. & Goerigk, L. Effect of the damping function in dispersion corrected density functional theory. *J. Comput. Chem.* **32,** 1456–1465 (2011).

23. Tang, W., Sanville, E. & Henkelman, G. A grid-based Bader analysis algorithm without lattice bias. *J. Phys. Condens. Matter* **21,** 84204 (2009).
